# How Artificial Intelligence Can Improve Our Understanding of the Genes Associated with Endometriosis: Natural Language Processing of the PubMed Database

**DOI:** 10.1155/2018/6217812

**Published:** 2018-03-20

**Authors:** J. Bouaziz, R. Mashiach, S. Cohen, A. Kedem, A. Baron, M. Zajicek, I. Feldman, D. Seidman, D. Soriano

**Affiliations:** ^1^Department of Obstetrics and Gynecology, The Chaim Sheba Medical Center, Ramat Gan, Israel; ^2^Artichoc Institue, 5 Alkalay, Tel Aviv, Israel; ^3^Department of Ophthalmology, Ichilov Hospital, Tel Aviv, Israel

## Abstract

Endometriosis is a disease characterized by the development of endometrial tissue outside the uterus, but its cause remains largely unknown. Numerous genes have been studied and proposed to help explain its pathogenesis. However, the large number of these candidate genes has made functional validation through experimental methodologies nearly impossible. Computational methods could provide a useful alternative for prioritizing those most likely to be susceptibility genes. Using artificial intelligence applied to text mining, this study analyzed the genes involved in the pathogenesis, development, and progression of endometriosis. The data extraction by text mining of the endometriosis-related genes in the PubMed database was based on natural language processing, and the data were filtered to remove false positives. Using data from the text mining and gene network information as input for the web-based tool, 15,207 endometriosis-related genes were ranked according to their score in the database. Characterization of the filtered gene set through gene ontology, pathway, and network analysis provided information about the numerous mechanisms hypothesized to be responsible for the establishment of ectopic endometrial tissue, as well as the migration, implantation, survival, and proliferation of ectopic endometrial cells. Finally, the human genome was scanned through various databases using filtered genes as a seed to determine novel genes that might also be involved in the pathogenesis of endometriosis but which have not yet been characterized. These genes could be promising candidates to serve as useful diagnostic biomarkers and therapeutic targets in the management of endometriosis.

## 1. Introduction

Endometriosis is a disease characterized by the development of endometrial tissue outside the uterus [1]. Symptoms include but are not limited to severe dysmenorrhea, pelvic pain, and reduced fertility [2]. The prevalence of endometriosis remains largely unknown; however, more than 180 million women worldwide are affected by the disease [3]. The diagnosis of endometriosis is challenging and often takes years, and the gold standard for diagnosis remains a visual inspection of the pelvis through laparoscopy and biopsy [4]. There is no simple biomarker with which to diagnose endometriosis [5]. Approximately 8–10% of women of all ages are affected, with approximately 20–25% in their fertile years [6].

The actual causes of the disease remain largely unclear, although numerous hypotheses have been proposed. Since 1927, it has been widely accepted that endometrial cells reach the peritoneal cavity by retrograde menstruation along the oviduct [7]. The main drawback to this theory is its inability to explain why menstrual debris is present in the peritoneal cavity of up to 90% of menstruating women, most of whom have no history of endometriosis. Other theories to explain its pathogenesis have been proposed, including the potential role of steroids, endometrial aberrations in the altered peritoneal environment, reduced immune surveillance, and increased angiogenic capacity [8]. Evidence is lacking, however, to better understand the disease evolution and to understand whether the previously mentioned processes are the cause or the effect of the disease.

A number of studies have shown that endometriosis could have a genetic basis [9, 10]. Higher disease rates were observed among relatives of patients with endometriosis than among controls in the general population [11] and in hospital-based samples [12]. The risk for an individual whose siblings have endometriosis is 15 times the risk for the general population. Other studies have shown concordance for the disorder among monozygotic twins [13] and increased concordance in monozygotic twins compared with dizygotic twins in a heritability estimate of 51% [10].

Familial aggregation has been observed in studies on nonhuman primates [14] and in studies on humans. Studies to determine the genetic and nongenetic basis of endometriosis using familial aggregation by phenotypic observations alone have assumed environmental interactions that are difficult to exclude. However, the role of genetics in the pathology of the disease can be tested directly using genome marker data through linkage or association studies without making these assumptions [15], although large samples are necessary for an accurate estimate.

Defining the disease pathways is one of the major goals of research into endometriosis and could ultimately help develop more efficient and earlier diagnostic methods and targeted treatments. The number of gene-mapping studies for endometriosis has increased in recent years as the contribution of genetic factors to the disease has become more widely accepted. There have also been dramatic advances in human genetics in the last few years, with a number of recent papers reporting genetic variations associated with other complex human disorders. These genetic studies are an important approach to define the causal pathways influencing endometriosis.

Artificial intelligence using text mining (TM) technology has recently been implemented [16], enabling the automated retrieval of all candidate genes for a specific disease from all published experimental results. Given the large number of candidate genes in endometriosis, implementing the standard functional validation of these candidate genes using experimental methodologies would no longer be possible without TM technology. By screening all articles published in the literature, TM can build an accurate list of candidate genes. Gene-focused TM is no simple task, however, and requires a number of steps to build an accurate list of candidate genes. A simple keyword search of articles would be infeasible because gene nomenclature and gene interconnections are more complex and vary among publications. Algorithms and deep learning processes are employed to perform two difficult tasks: gene mention recognition and gene normalization. Computational methods could also help provide a useful alternative for determining the pathways and the enriched networks of related genes. Once these steps have been performed, genes can be ranked according to their relevance in the disease pathway, establishing* candidate gene prioritization*.

Although there has been a dramatic paradigm shift in molecular biology from single genes or proteins to genomics or proteomics, studies addressing endometriosis using whole-genome expression have been scarce. However, biomedical research is a field in which TM can be rigorously applied to obtain relevant information, developing a database that can help in understanding and analyzing gene-related diseases and the interactions among molecules and proteins.

In the current study, we analyzed the results of the TM analysis of PubMed literature on genes involved in the pathogenesis of endometriosis. We systematically characterized the expression of endometriosis-associated genes by mining data from the PubMed document retrieval system. We also used bioinformatics to analyze the functions, pathways, and networks of relevant candidate genes, seed genes, and novel genes.

## 2. Materials and Methods

### 2.1. Text Mining

The TM data extraction was based on natural language processing (NLP) [17], which can be defined as a computer program's ability to understand spoken and written language and is a component of artificial intelligence [18].

The PubMed database was used as a source of publications for the TM process. The database was searched with the keywords “endometriosis and genes” and “endometriosis and genetic”. The relevant publications were retrieved in extensible markup language (XML) format in order to make the information extraction more precise, with content enclosed within XML tag pairs [19]. The titles and abstracts of each article were converted into the PubTator format [20] through a custom Perl script.

For the TM, we employed the GNormPlus system [21], which contains two modules (gene mention recognition and gene normalization) essential for understanding the specific and complex nature of TM when focusing on genes.

### 2.2. Gene Mention Recognition

Currently, gene nomenclature cannot be standardized due to the lack of consensus, leading to disarray, given that gene names are used based on the situation and requirement. Given that there are approximately a million genes, naming the genes is no easy task. Finding a gene name using TM is therefore labor intensive and equivalent to searching for a name in a newspaper, a process known as name entity recognition (NER), which has become an issue of concern among researchers. NER techniques have developed and improved in recent years; however, the continuous development of genes and the frequent renaming of previously named genes further complicate the NER task, ultimately hindering the biomedical domain research process.

The GNormPlus system performed the gene mention recognition using the CRF++ library, an open source implementation of conditional random fields (CRF) for segmenting and labeling sequential data. CRF++ can be used for various tasks, including NER, data extraction, and text chunking. CRF, in contrast, involves statistical modeling and is most commonly used for predicting and analyzing patterns and for machine learning. The most important use of CRF is to assist in understanding the subject in context.

### 2.3. Gene Normalization

Genes normalization is the process of mapping a name in the text to a unique identifier that is then attributed to a DNA series in a particular chromosome. This series of tasks involves several aspects, including species assignation and species-specific gene normalization, which is a complicated challenge, involving* gene mention variation*,* orthologous gene ambiguity,* and* intraspecies gene ambiguity*.

GeNorm, an algorithm for determining housekeeping genes from candidate reference genes, was employed during the gene normalization process.

Finally, all curated entries were summarized, and a full list of genes associated with endometriosis was compiled.

### 2.4. Analysis of Gene Ontology, Pathways, and Networks

Gene ontology (GO) is the bioinformatics step for unifying the representation of genes and gene products among species. The core aims of gene ontology are as follows:

(1) Manage and construct a standard vocabulary for genes and their products.

(2) Annotate the gene and its products and integrate and disseminate the product's attributes.

(3) Provide easy access and availability to the tools to access the information offered by the project. Enable the enrichment analysis by providing the GO for the functional interpretation of experimental data.

GO is a framework for the biological model that defines the concepts and classes employed to describe the functions of genes. GO also defines the functions and relationships between the classes and concepts and classifies these functions into the following three factors:

(1) Molecular function: the activities in the molecules of a gene product

(2) Cellular component: an entity in which there are active gene products

(3) Biological process: processes developed from the activities conducted or occurring from multiple gene products

We employed Bingo 2.3 software with the GOSlim database for the GO analysis (Gene Ontology Consortium 2004) [22]. For the enrichment, we performed a hypergeometric test, followed by a customized Bonferroni multiple test correction. The *p* value, adjusted to 0.005, was used as the significant threshold to identify the enriched GO terms. The R package word cloud was employed to generate the word cloud, a method for confirming that the genes we are using are relevant to the disorder under study, as the cloud describes the disorder or the disorder's symptoms.

The pathway enrichment analysis was performed using the DAVID tools [23]. Network creation was performed using the STRING database 10.5, which was then loaded into Cytoscape software for visualization and analysis.

### 2.5. Candidate Gene Prioritization

Prioritization consists of ranking genes according to their relevance in the disease pathways. We employed Phenolyzer software [24] for the gene prioritization throughout the human genome. Phenolyzer prioritizes disease genes based on any disease/phenotype terms used as input. The order is based on a score that represents the candidate gene's probability ranking, which is highly dependent on its frequency and the number of candidate genes under consideration. In this study, all genes with a score of 0.75 or above were selected, resulting in a total of 27 candidate genes.

## 3. Results

### 3.1. List of Endometriosis Genes

**Figure 1:**
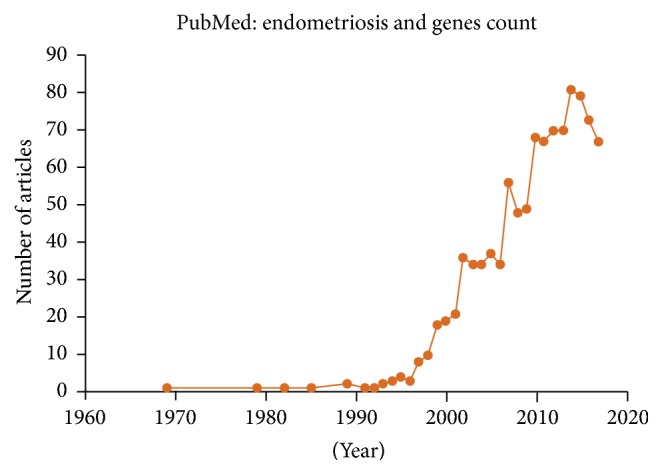
PubMed articles related to the genetic mechanisms of endometriosis.

After searching for the previously mentioned keywords in the PubMed database, we retrieved a total of 999 articles (from 1969 to 2017). We observed that the number of articles related to the genetic mechanisms of endometriosis has been steadily increasing since 1969, as shown in Figure 1.

**Figure 2:**
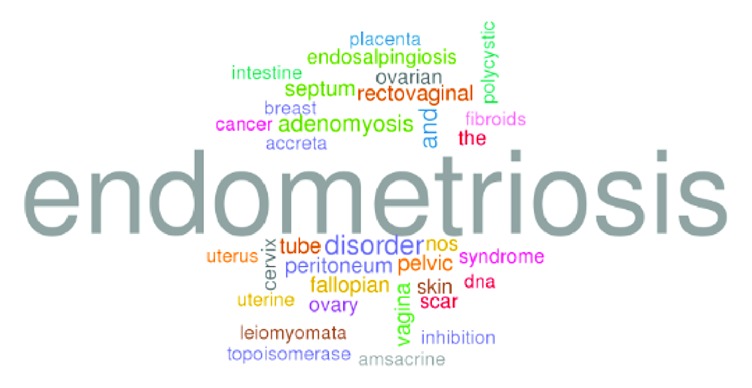
Word cloud of enriched gene ontology terms among the endometriosis-associated candidate genes.

**Figure 3:**
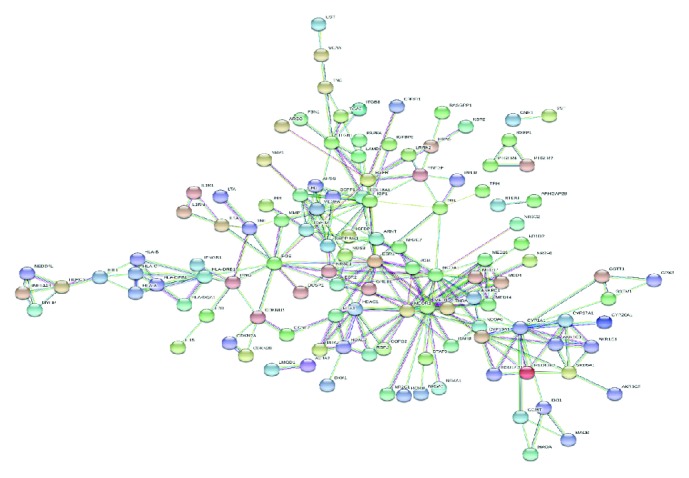
Network structure underlying all endometriosis-related genes. The edges represent interactions, whereas the nodes represent the genes.

**Figure 4:**
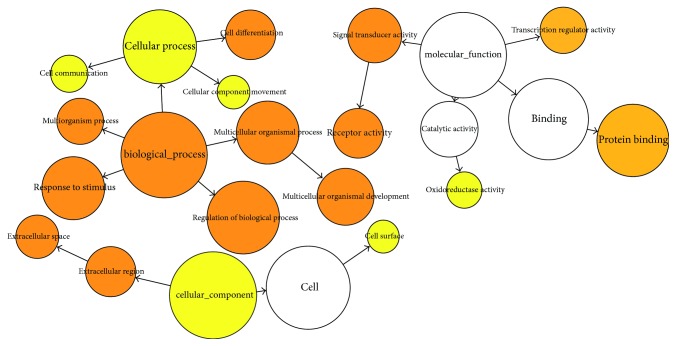
Network structure underlying all endometriosis-related genes. The edges represent interactions, whereas the nodes represent the genes.

The titles and abstracts of these articles were retrieved and processed through a computational pipeline through text mining, which yielded a total of 724 genes. The text mining results were then filtered by Phenolyzer software [24]. The likelihood that a specific gene was generated by chance was calculated and then corrected. A total of 203 genes reached the statistical significance threshold (*p* = .05) and were used to generate the word cloud (Figure 2), the network (Figure 3), and the pathway enrichment (Figure 4).

### 3.2. Endometriosis-Associated Gene Characterization

The 203 genes significantly associated with endometriosis were then tested for the functional enrichment categories, including the pathways and gene ontology terms. All candidate genes were functionally characterized based on GOSlim annotations, using the Bingo tool. Enriched GO terms are classified according to biological process (BP), molecular function (MF), and cellular component (CC) (Figure 3). The enriched GO terms included the multiorganism process (*p* = 1.90*E* − 15), response to stimuli (*p* = 6.01*E* − 14), signal transducer activity (*p* = 7.70*E* − 13), regulation of the biological process (*p* = 1.3071*E* − 12), extracellular space (*p* = 8.00*E* − 12), cell differentiation (*p* = 6.69*E* − 10), receptor activity (*p* = 9.47*E* − 10), multicellular organism development (*p* = 3.33*E* − 09), multicellular organism process (*p* = 3.96*E* − 09), biological process (*p* = 1.10*E* − 07), extracellular region (*p* = 2.01*E* − 07), protein binding (*p* = 3.10*E* − 06), transcription regulator activity (*p* = 3.64*E* − 06), cell surface (*p* = 2.32*E* − 04), oxidoreductase activity (*p* = 8.83*E* − 04), cellular component (*p* = 1.07*E* − 03), cellular process (*p* = 2.02*E* − 03), cellular component movement (*p* = 2.96*E* − 03), and cell communication (*p* = 3.48*E* − 03).

In the MF category, 8 terms were found to be enriched: signal transducer activity (*p* = 7.6972*E* − 13), receptor activity (*p* = 9.4729*E* − 10), transcription regulator activity (*p* = 3.6422*E* − 6), protein binding (*p* = 3.0982*E* − 6), binding (*p* = 9.6394*E* − 3), oxidoreductase activity (*p* = 8.8272*E* − 4), molecular function (*p* = 3.5159*E* − 2), and electron carrier activity (*p* = 2.2095*E* − 2). The CC category had 4 enriched terms: extracellular region (*p* = 2.0120*E* − 7), extracellular space (*p* = 7.9967*E* − 12), cell surface (*p* = 2.3183*E* − 4), and cellular component (*p* = 1.0712*E* − 3) (Figure 4).

### 3.3. Gene Prioritization

**Figure 5:**
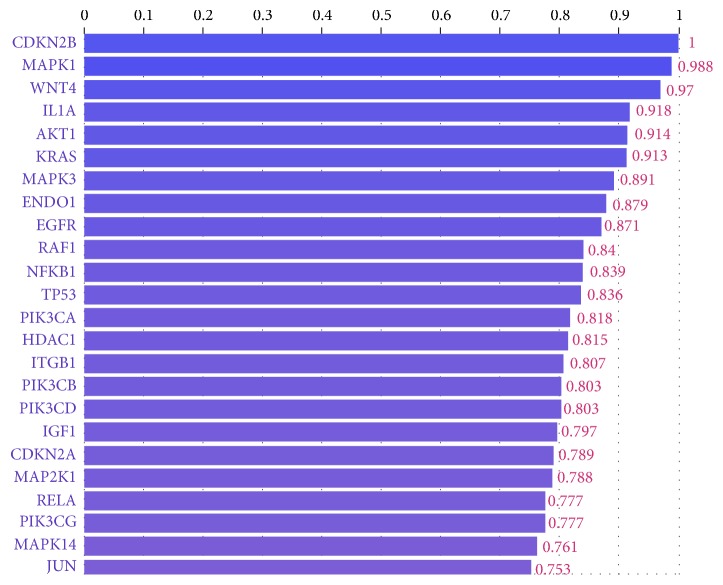
A list of the first 24 prioritized genes with their scores.

Genes involved in the same disease have been shown to share annotations in the InterPro GO and databases. In addition, genes on the same pathway have been found to have a high degree of sequence similarity, indicating functional importance for a particular disease. In this study, gene prioritization was performed with the web-based Phenolyzer software, which provides superior performance over other competing methods for prioritizing Mendelian and complex disease genes, based on disease or phenotype terms entered as free text. The 203 genes found to be significantly associated with endometriosis were used as a training set. Phenolyzer scanned the whole human genome and globally ranked the generated genes according to their scores. The graph in Figure 5 represents the first 24 prioritized genes whose score was above 0.75 (measured on a scale of 0 to 1).

The prioritized genes included novel genes that relate to the candidate genes (seed genes). Novel genes are those not found directly and specifically in the screened literature, which does not imply that the genes have not been studied, just that they have not been mentioned in the abstracts.

**Figure 6:**
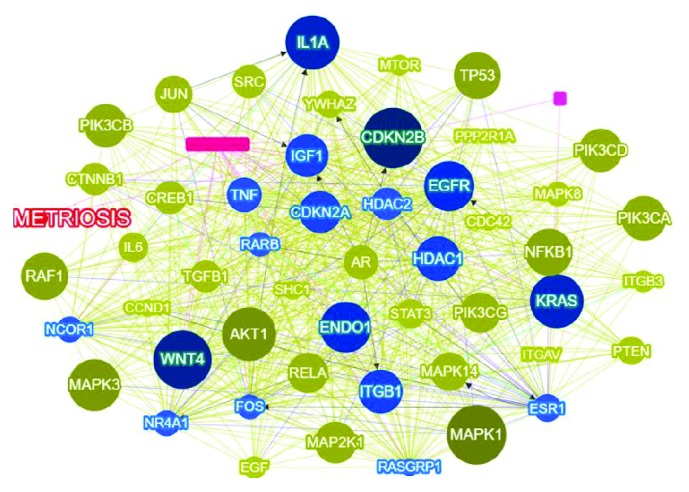
Interaction of endometriosis-related candidate and novel genes. Blue circles represent seed genes, whereas green circles represent novel genes.

The first 9 novel genes (and their scores) included MAPK1 (0.9883), AKT1 (0.9139), PRKCA (0.8962), CREBBP (0.8924), MAPK3 (0.8914), PRKACA (0.8566), PIK3R2 (0.8424), EP300 (0.8424), and RAF1 (0.8400). The network in Figure 6 represents the interaction of novel genes with the seed genes in the pathogenesis of endometriosis.

## 4. Discussion

In recent years, there has been a steady increase in the number of articles on endometriosis. Numerous genes and pathways believed to be involved in the pathogenesis of this disease have been described. However, these efforts have been unable to fully describe the mechanisms underlying endometriosis. Our study was therefore aimed at summarizing the most important genes already published and discovering new ones that have not yet been clearly linked to endometriosis. The interpretation of TM results is typically based on the number of publications in which a particular gene is mentioned. The importance of this fact cannot be ignored because the more a gene is mentioned, the more useful it is to biologists. The drawback of TM is that it treats genes as separate entities, as they relate to one another in biological systems. However, Berggård et al. (2017) demonstrated that, in many cases, gene products (proteins) typically combine to produce protein complexes that perform a combined function instead of the genes' individual functions. Thus, a gene has functional relevance for its interacting partners, thereby opening up the possibility of integrating interaction information for gene prioritization. In this study, we employed the STRING database to construct a genome-wide gene network using up-to-date interaction data from the database. The threshold was set at 0.9 for the combined score, thereby obtaining a gene network consisting of 194 nodes connected through 243 edges, proving once again the complex and multifactorial nature of endometriosis. Finding a unique protein or gene to act as the diagnostic indicator is overly optimistic; however, the possibility of finding a protein complex should be examined.

Using TM data and gene network information as input, Phenolyzer prioritized the endometriosis-related genes. After the Phenolyzer was fed the seed genes, the software produced a list of 15207 genes, arranged by score. These many genes cannot be easily studied using traditional genetic investigation methods or by microarray. Using artificial intelligence and various tools to determine gene functionality, we were able to list the most relevant genes.

The top 5 genes selected by Phenolyzer (with a score of 0.9–1.0) were as follows:

(i) Cyclin-dependent kinase inhibitor 2B (CDKN2B), whose rs1537377 polymorphism showed significant associations with endometriosis [25], was found in other studies [5, 26] to play a key role in the pathogenesis.

(ii) Mitogen-activated protein kinase 1 (MAPK1) encodes a member of the MAP kinase family. MAP kinases act as an integration point for multiple biochemical signals and are involved in a wide variety of cellular processes, including proliferation, differentiation, transcription regulation, and development. MAPK1 was considered a novel gene in the study.

(iii) Wnt family member 4 (WNT4) belongs to a family of structurally related genes that encode secreted signaling proteins. Studies have suggested that rs16826658 and rs3820282 polymorphisms on the WNT4 gene are associated with the pathogenesis of endometriosis in infertile women (Mafra 2015).

(iv) Interleukin 1 alpha (IL1A) is a pleiotropic cytokine involved in various immune responses, inflammatory processes, and hematopoiesis. The cytokine is produced by macrophages and monocytes as a proprotein, which is proteolytically processed and released in response to cell injury and thus induces apoptosis. Sapkota et al. showed that rs6542095 SNP at the IL1A locus was significantly associated with the pathogenesis of endometriosis.

(v) Serine/threonine kinase 1 (AKT1) encodes serine-threonine protein kinase, which is catalytically inactive in serum-starved primary and immortalized fibroblasts. In a study comparing the expression levels of relevant angiogenesis-related genes in the eutopic endometrium of women with and without endometriosis, high AKT1 levels were observed as compared with controls (Laudanski et al. 2014).

(vi) The KRAS proto-oncogene (KRAS) provides instructions for synthesizing the K-Ras protein, which is significantly involved in regulating cell division. As part of the RAS/MAPK signaling pathway, this protein relays important signals from outside the cell to the cell's nucleus, instructing the cell to grow and divide or to mature and assume specialized functions. Together with SIRT1 and BCL6, KRAS was overexpressed in the eutopic endometrium of women with endometriosis (Yoo et al. 2017). KRAS therefore likely participates in the pathogenesis of endometriosis.

This panel of high-scoring genes could serve as a useful diagnostic marker and for managing endometriosis. MAPK1 is considered a novel gene and received a very high score of 0.988, which is rare for a novel gene.

The hypothesis that the MAPK is linked to the pathogenesis of endometriosis is not new and has already been investigated. However, our TM process could not find any abstract that specifically mentioned the role of MAPK1 in endometriosis. However, MAPK1's high score of 0.988 indicates the importance of this gene in the pathogenesis of endometriosis.

Chernigovskaya et al. (2017) found that activity due to its product could be the cause of neurotransmitter system function abnormalities in mice. Our results agree with those of a study by Santulli et al. [27], who considered that MAPK could be a good target for endometriosis treatment.

Another method for improving our understanding of a complex disease is to identify the genetic factors that underlie the disease risk through genome-wide association studies, which have the advantage of bypassing the weaknesses of candidate associations based only on a priori biological hypotheses. Uimari et al. conducted such a study with the largest series to date, consisting of 3194 cases of surgically confirmed endometriosis and 7060 controls, with genotype data that was imputed to the latest 1000 Genomes Phase 3 reference panel. The authors found that the top pathways significantly associated with all endometriosis stages included several MAPK-related pathways, one of which was MAPK1. Although our results are less detailed than those of the Uimari et al. study, our results are similar, and our study was cost effective and faster to perform.

## 5. Conclusion

The use of artificial intelligence through TM combined with bioinformatics techniques (including gene recognition, gene normalization, and gene ontology) can help us understand gene networks and pathways, candidate genes, and novel genes. The rapid improvement in algorithm and machine-learning efficiency is of great interest in helping researchers focus on the relevant candidates and novel genes for endometriosis before performing* in vivo *studies or microarrays. The top 6 genes associated with endometriosis found in our study are CDKNB2, MAPK1, WNT4, ILA, AKT1, and KRAS. Our results agree with those from microarray and genome-wide analysis studies, a highly encouraging outcome.
